# Skin manifestations after bariatric surgery

**DOI:** 10.1186/s12895-020-00120-z

**Published:** 2020-12-09

**Authors:** Yada Itthipanichpong, Wilawan Damkerngsuntorn, Natsinee Tangkijngamvong, Suthep Udomsawaengsup, Patchaya Boonchayaanant, Chanat Kumtornrut, Stephen J. Kerr, Pravit Asawanonda, Pawinee Rerknimitr

**Affiliations:** 1 Division of Dermatology, Department of Medicine, Faculty of Medicine, Skin and Allergy Research Unit, Chulalongkorn University, 1873 Rama IV Road, Pathumwan, Bangkok, 10330 Thailand; 2 Department of Surgery, Faculty of Medicine, Chulalongkorn University, Bangkok, Thailand; 3 Division of Endocrinology and Metabolism, Faculty of Medicine, Chulalongkorn University, Bangkok, Thailand; 4 Center for Excellence in Biostatistics, Faculty of Medicine, Chulalongkorn University, Bangkok, Thailand

**Keywords:** Skin, Cutaneous sign, Obesity, Bariatric surgery, Weight loss

## Abstract

**Background:**

Skin signs observed in morbid obesity may change as the weight reduces, especially post-bariatric surgery (BaS). Data concerning the skin findings exclusively in post-BaS patients remain limited.

**Methods:**

Seventy post-BaS patients were examined for cutaneous abnormalities. The patients were divided into those with successful weight loss (% excessive body weight loss (EBWL) of at least 50%) and a non-successful group (%EBWL < 50%).

**Results:**

Forty-six patients with successful weight loss demonstrated a significantly lower prevalence of acanthosis nigricans on the neck, axillae and inguinal areas, keratosis pilaris (KP) and pebble fingers. However, a higher prevalence of alopecia was observed. After adjustment with patients’ factors, KP (adjusted odds ratio (aOR) = 0.21, 95%CI 0.06–0.74, *p* = 0.02) and pebble fingers (aOR = 0.09, 95%CI 0.01–0.89, *p* = 0.04) remained significantly less likely in patients with successful weight loss. Laboratory results comparing pre- and post-surgery values revealed significant decreases in fasting plasma glucose, hemoglobin A1c, and triglyceride and an increase of high-density lipoproteins in both groups. However, significant decreases of liver aminotransferases (AST and ALT) were observed only in the successful group (*p* = 0.04, 0.003). Nonetheless, a decrease in vitamin B12 (*p* = 0.01) was observed in the successful group.

**Conclusion:**

Weight loss after BaS provided an improvement for metabolic profiles. Successful weight reduction resulted in better skin improvement. However, nutritional supplements may be necessary.

**Trial registration:**

Thai Clinical Trials Registry TCTR20171003002. Registered October 3. 2017, retrospectively registered.

## Background

Obesity represents one of the major health concerns on a global scale. Substantial increases in the prevalence of patients with obesity has raised the public awareness over decades. The prevalence of overweight individuals (body mass index (BMI) ≥ 25 kg/m^2^) was reported to be as high as 40% of the total world population [1]. Obesity has various impacts on the skin, including changes in skin physiology, myriads of skin manifestations and aggravation of skin diseases.

Skin signs associated with obesity may be divided into those that occur as a result of insulin resistance such as acanthosis nigricans (AN) [2–4] acrochordons [3, 4], and keratosis pilaris (KP) [3–5]. Additionally, mechanical effects may give rise to plantar hyperkeratosis, striae distensae, adiposis dolorosa, and chronic venous insufficiency [3, 4]. Skin infections, i.e. candidiasis and the inflammatory skin disorders namely psoriasis and hidradenitis suppurativa are observed at higher frequency and severity in patients with obesity [3, 4, 6]. Finally, skin manifestations due to metabolic derangements such as gouty tophi are also seen [3, 4].

AN is characterized by symmetric velvety hyperpigmented plaques. The common locations are on the neck, axillae, inguinal, inframammary, and genital regions [7, 8]. AN is reported to be found in as high as 74% in patients with obesity [9]. Acrochordons or skin tags are pedunculated soft brownish papules. They are commonly detected together with AN on the neck, axillae, and inguinal area [10]. KP is manifested as multiple small, perifollicular, spiny papules on the extensor aspect of the extremities. Pebble fingers, or Huntley’s papules are classically described in association with diabetes mellitus (DM) type 2 in patients with obesity [8, 11]. They usually present as asymptomatic grouped, multiple minute papules, which may coalesce into confluent plaques. Common locations are the dorsum of the hand, knuckles, and periungual areas. It is believed that pebble fingers represent a variation of diabetic thick skin [8].

Treatments and interventions to induce weight loss are of paramount importance, especially for patients with morbid obesity (BMI ≥ 40 kg/m^2^) [12]. Bariatric surgery is one of the most effective long-term weight reduction therapies. Success of bariatric surgery is defined as whether at least 50% excessive body weight loss (EBWL) is achieved [13]. Bariatric surgery may lead to 60–80% EBWL in the first year and stabilize at 50–60% in 80% of gastric bypass patients [14].

Moreover, successful bariatric surgery has demonstrated a wide range of health benefits, including the skin and overall medical conditions. Improvements of obesity-associated complications namely DM, hypertension, dyslipidemia, and obstructive sleep apnea have been documented [12, 14, 15]. In addition, bariatric surgery has both positive and negative impacts on the skin. There are reports of improvements in many skin conditions such as psoriasis [16, 17], intertrigo, hidradenitis suppurativa and ulcerated necrobiosis lipoidica diabeticorum [17, 18]. Conversely, the occurrence of adverse skin conditions, mostly involving nutritional deficiency, for example, of iron, folic acid, vitamin and trace elements, has been reported [17–20]. Furthermore, massive weight loss also contributes to skin laxity [21].

Nonetheless, information concerning the skin signs exclusively seen in post-bariatric surgery patients remain limited. To our knowledge, there is no study comparing the skin signs in those with successful versus non-successful weight loss. Therefore, we conducted this cross-sectional study to investigate the skin signs, skin diseases, and laboratory values in post-bariatric surgery patients, in an attempt to shed some light in this area that may be beneficial for clinical practice and counseling patients.

## Methods

### Study design and ethical consideration

This cross-sectional study was conducted at King Chulalongkorn Memorial Hospital, Bangkok, Thailand from February to November 2017. The trial protocol was approved by the local Institutional Review Board, the approval number 633/60. The trial was registered with Thai Clinical Trials Registry, TCTR number 20171003002. Written informed consent was obtained from all participants. The present study adheres to CONSORT guidelines.

### Study subjects

The inclusion criteria were as follows: patients aged more than 18 years, diagnosed with morbid obesity (BMI ≥ 40 kg/m^2^) who had undergone a bariatric surgery. Exclusion criteria were patients who were not willing to participate or have skin conditions treated 6 months prior to the examination.

### Study protocol

Consecutive cases of post-bariatric surgery patients were interviewed and thoroughly examined for the presence of any skin abnormalities by certified dermatologists. The skin examination was conducted once in all patients at a post-surgical visit, therefore the skin findings were obtained only at one point in time. However, information regarding the types of bariatric surgery, clinical data and laboratory values (at baseline, immediately before- and at 6 months, or later dates, where applicable after the surgery) were extracted from the medical records. The diagnosis of alopecia was based on the history of increase hair shedding and a thorough hair and scalp examination was then performed. The patients were categorized into two groups according to their weight loss status. The results of surgery were defined as successful or non-successful, as detailed above. %EBWL was calculated in accordance with previous reports; [(pre-operative BMI – follow-up BMI)/(pre-operative BMI – Ideal Body Weight (IBW))] × 100. Ideal body weight was based on a BMI of 25 kg/m^2^ [13, 15, 22].

### Statistical analysis

Given the lack of information on skin signs in post-bariatric patients, we based our sample size on the error around potential skin condition prevalence estimates of approximately 25% in post-bariatric patients. Enrolling 72 patients would allow estimation of the prevalence with an error of approximately +/− 10%.

Statistical analysis was conducted using Stata 15.1 (Statacorp, College Station, TX, USA). Patients demographics and bariatric surgery data were described as mean (standard deviation [SD]) for continuous variables and N (%) for categorical variables. The prevalence of skin signs and diseases was determined based on the presence or absence and described as N (%), and the odds ratios (OR) of each sign in those with successful versus non-successful surgery was calculated using logistic regression. Where we found evidence of a significantly increased or decreased odds in those with successful surgery, we adjusted these for age, sex, and underlying diseases and finally reported these adjusted odds ratio (aOR), 95% confident interval (95% CI), and *p*-values. Changes in laboratory results was compared between baseline and a more than 6-month post bariatric surgery using paired-t test and reported as mean difference (95%CI) both within and between weight-loss groups. The comparison of the percentage change of the lab results between successful and non-successful group were compared using an independent-t test.

## Results

Seventy patients completed the study. The mean age was 41(9.60) years. A female predominance was observed with 46 (65.7%) females and 24 (34.3%) males. There were 46 (65.7%) patients who attained successful weight loss and 24 (34.3%) with non-successful weight loss. Baseline demographic data of the patients are shown in Table 1. No statistically significant differences were observed between these 2 groups.

**Table 1:** Demographic data of seventy post-bariatric surgery patients; overall and according to weight loss status

	Total patients (***N*** = 70)	Non-successful weight loss patients (***N*** = 24, 34.3%)	Successful weight loss patients (***N*** = 46, 65.7%)
Mean age (SD)	41 (9.6)	40 (8.7)	42 (10)
Sex			
Male (%)	24 (34.3%)	11 (45.8%)	13 (28.3%)
Female (%)	46 (65.7%)	13 (54.2%)	33 (71.7%)
Bariatric surgery			
RYGB^a^	48 (68.6%)	17 (70.8%)	31 (67.4%)
SG^a^	22 (31.4%)	7 (29.2%)	15 (32.6%)
Mean (SD) months after surgery	14.5 (20)	9.98 (14.8)	18 (20.8)
Mean (SD) preoperative weight	131.38 (29.85)	137.22 (28.82)	128.33 (30.24)
Mean (SD) preoperative BMI	43.38 (9.68)	50.17 (9.54)	47.44 (9.73)
Mean (SD) current weight	94.83 (23.9)	114.7 (24.88)	84.47 (15.46)
Mean (SD) current BMI^a^	34.9 (8.14)	42 (8.43)	31.3 (5.03)
Mean (SD) total weight reduction percentage	27.31 (27.37)	16.2 (6.95)	33.11 (7.81)
Mean (SD) EBWL^a^ percentage	60.7 (26.2)	32.9 (13)	75.2 (18.4)
Diet modification			
Yes (%)	59 (84.3%)	21 (87.5%)	38 (82.6%)
No (%)	11 (15.7%)	3 (12.5%)	8 (17.4%)
Underlying diseases			
Hypertension, n (%)	42 (60%)	15 (62.5%)	27 (58.7%)
Dyslipidemia, n (%)	38 (54.3%)	16 (66.7%)	22 (47.8%)
Diabetes, n (%)	37 (52.9%)	15 (62.5%)	22 (47.8%)
PCOS^a^, n (%)	19 (27.1%)	5 (20.8%)	14 (30.4%)
Others^b^, n (%)	100 (142.9%)	37 (154.7%)	74 (158.7%)

^a^*BMI* body mass index, *EBWL percentage *excess body weight loss percentage, calculated by [(pre-operative BMI – follow-up BMI)/ (pre-operative BMI – Ideal Body Weight (IBW))] × 100. Ideal body weight was based on a BMI of 25 kg/m^2^, *RYGB* Roux-en-Y gastric bypass, *PCOS* polycystic ovarian syndrome, *SG* sleeve gastrectomy; (kg/m2)

^b^Others underlying diseases are referred to coronary artery diseases, obstructive sleep apnea (OSA), non-alcoholic steatohepatitis (NASH)/non-alcoholic fatty liver disease (NAFLD), kidney disease, inflammatory diseases, hypothyroidism, malignancy, hyperuricemia

**Fig. 1:**
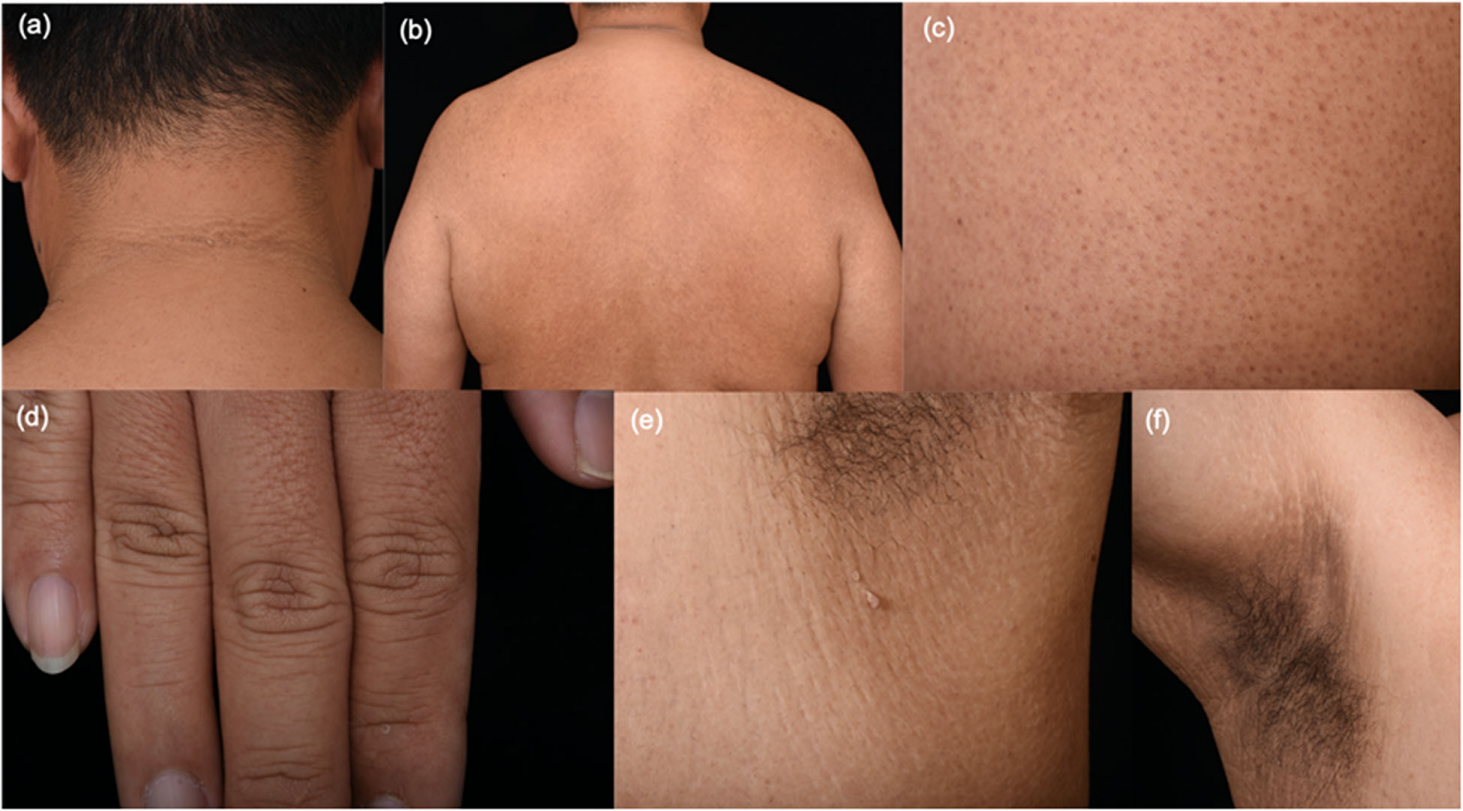
Acanthosis nigricans and acrochordon on the neck (**a**); keratosis pilaris on the back (**b**); keratosis pilaris on the arm (**c**); pebble fingers (**d**); intertrigo and acrochordons on left axilla (**e**); intertrigo and striae on right axilla (**f**) in a patient with 48.9%EBWL

The presence of the skin manifestations in the two groups are shown in Table 2. Patients in the successful weight loss group demonstrated a statistically significantly lower prevalence of AN on the neck, axillae and inguinal area, KP and pebble fingers, compared to the non-successful counterparts. However, the higher prevalence of alopecia was observed. The number of other skin manifestations did not differ between the groups. After an adjustment for age, sex, and underlying diseases, KP (adjusted OR = 0.21, 95%CI 0.06–0.74, *p* = 0.015) and pebble fingers (adjusted OR = 0.09, 95%CI 0.01–0.89, *p* = 0.039) remained statistically significant. Some of the skin manifestations are shown in Fig. 1.

**Table 2:** Logistic regression results showing odds and adjusted* odds ratios of skin manifestations in patients who had successful versus non-successful weight loss post bariatric surgery

			Univariate		Adjusted	
Skin manifestations	Non-successful weight loss patients (***N*** = 24)	Successful weight loss patients (***N*** = 46)	OR (95%CI)	***p***-value*	Adjusted OR (95%CI)^**a**^	Adjusted ***p***-value*
Hair						
Alopecia (%)	10 (41.67%)	31 (67.39%)	2.89 (1.04,8.02)	0.04*	2.75 (0.88, 8.57)	0.08
Skin						
Psoriasis (%)	0 (0%)	2 (4.35%)	1 (−)	–	–	–
Diagonal earlobe creases (%)	1 (4.17%)	1 (2.17%)	0.51 (0.03, 8.55)	0.64	–	–
Acanthosis nigricans (%)	15 (62.5%)	19 (41.3%)	0.42 (0.15, 1.16)	0.10	–	–
AN-neck (%)	15 (62.5%)	17 (36.96%)	0.35 (0.13,0.98)	0.045*	0.33 (0.09, 1.17)	0.09
AN-axillae (%)	11 (45.83%)	9 (19.57%)	0.29 (0.1, 0.85)	0.03*	0.37 (0.11, 1.27)	0.11
AN-antecubital fossa (%)	2 (8.33%)	2 (4.35%)	0.5 (0.07, 3.79)	0.50	–	–
AN-inguinal areas (%)	6 (25%)	3 (6.52%)	0.21 (0.05, 0.93)	0.04*	0.21 (0.04,1.21)	0.08
AN-inframammary areas (%)	1 (4.17%)	0 (0%)	1 (−)	–	–	–
Acrochordons/skin tags (%)	11 (45.83%)	24 (52.17%)	1.29 (0.48, 3.47)	0.62	–	–
Acrochordons/skin tags -neck (%)	8 (33.33%)	22 (47.83%)	1.83 (0.66, 5.12)	0.25	–	–
Acrochordons/skin tags-axillae (%)	3 (12.5%)	9 (19.57%)	1.7 (0.41, 6.99)	0.46		
Acrochordons/skin tags-inguinal areas (%)	0 (0%)	2 (4.35%)	–	–	–	–
Acrochordons/skin tags-eyelid (%)	1 (4.17%)	1 (2.17%)	0.51 (0.03, 8.55)	0.64	–	–
Acrochordons/skin tags-trunk (%)	5 (20.83%)	6 (13.04%)	0.57 (0.15,2.10)	0.40	–	–
Acrochordons/skin tags-inframammary areas (%)	1 (4.17%)	0 (0%)	1 (−)	–	–	–
KP (%)	14 (58.33%)	12 (26.09%)	0.25 (0.09, 0.72)	0.01*	0.21 (0.06, 0.74)	0.02*
Pebble fingers (%)	5 (20.83%)	1 (2.17%)	0.08 (0.01, 0.77)	0.03*	0.09 (0.01, 0.89)	0.04*
Striae distensae (%)	16 (66.67%)	27 (58.7%)	0.71 (0.25, 1.99)	0.52	–	–
Folliculitis (%)	2 (8.33%)	2 (4.35%)	0.5 (0.07, 3.8)	0.50	–	–
Intertrigo (%)	7 (29.17%)	12 (26.09%)	0.86 (0.29, 2.57)	0.78	–	–
Chronic venous disease manifestations (%)	9 (37.5%)	11 (23.91%)	0.52 (0.18, 1.52)	0.24	–	–
Plantar hyperkeratosis (%)	19 (79.17%)	27 (58.70%)	0.37 (0.12, 1.18)	0.09	–	–

*AN* acanthosis nigricans, *KP* keratosis pilaris

*p < 0.05

^a^ Adjusted for age, sex, and underlying diseases (i.e. hypertension, dyslipidemia, diabetes mellitus, and polycystic ovarian syndrome)

There were 41 (58.6%) patients with alopecia in this present study; 31 from the successful weight loss group and 10 from the non-successful. All of the cases were non-scarring alopecia. The diagnosis of telogen effluvium (diffuse and excessive shedding more than 70–100 hairs per day and clinical examination showed diffuse hair thinning) was made in 14 patients while a pattern hair loss compatible with androgenetic alopecia was found in 34 patients. Seven patients have both diffuse and pattern hair loss. Hair pulling test was found positive (≥3 hairs) in 3 patients. In the diffuse hair loss group, the mean onset of hair loss was 3.07 ± 1.2 months after the surgery.

In addition to the classic skin manifestations reported in the patients with obesity, the following mucocutaneous signs were observed in our patients; pigmented fungiform papillae (*N* = 1), lichen amyloidosis on the extremities (*N* = 3 patients, 2 in the successful weight loss group and 1 in the other), hypertrophic scar (*N* = 1), keloid (*N* = 1), en coup de sabre-type morphea (*N* = 1), chronic paronychia (*N* = 1), callus (*N* = 1), and fibrous papules of the nose (*N* = 1). However, the prevalence of all of these signs was low, and showed no differences between the two groups.

The within-group laboratory results comparing pre- and post-surgery values are shown in Table 3. Both successful and non-successful weight loss groups showed statistically significant decreases of white blood cells (WBC) (*p* < 0.001 and 0.016 in the successful and non-successful groups, respectively), as well as fasting plasma glucose (FPG) (*p* = 0.005 and 0.01), HbA1C (*p* = 0.003 and < 0.001) and triglyceride (TG) (*p* = 0.005 and 0.034). An increase of high-density lipoproteins (HDL) was also observed in both groups (*p* = 0.001 and 0.034). Additionally, only the successful weight loss group exhibited a statistically significant decreases of aspartate aminotransferase (AST) (*p* = 0.043), alanine aminotransferase (ALT) (*p* = 0.003), vitamin B12 (*p* = 0.048), and total iron binding capacity (TIBC) (*p* = 0.001) and an increase in total cholesterol (TC) (*p* = 0.037) after the surgery.

**Table 3:** Laboratory changes from baseline to > six months, categorized according to weight loss status

Parameter	Non-successful weight loss patients			Successful weight loss patients			***P***. value (mean changes from baseline to > 6 months between weight loss groups)
	n	Mean changes from baseline to > 6 months (95% CI)	***p***. value	n	Mean changes from baseline to > 6 months (95% CI)	***p***. value	
Hb	14	−0.22 (− 0.83, 0.38)	0.44	33	− 0.35 (− 0.71, 0.004)	0.05	0.69
Plt (×10^3^)	14	−19.29 (− 41.58, 3.006)	0.08	33	−35.12 (−51.61, − 18.63)	< 0.001*	0.27
WBC (× 10^3^)	14	− 1.5 (−2.67, − 0.33)	0.02*	33	−2.88 (− 3.68, − 2.09)	< 0.001*	0.05
FPG	13	−18.08 (−30.9, − 5.25)	0.01*	29	−7.76 (− 12.97,-2.55)	0.005*	0.07
HbA1C	12	−0.875 (−1.17, − 0.58)	< 0.001*	22	− 1.02 (− 1.66,-0.39)	0.003*	0.73
Cr	13	0.04 (−0.07, 0.15)	0.44	33	0.25 (−0.40, 0.90)	0.44	0.68
AST	14	−5.5 (−15.46,4,46)	0.25	33	−7.24 (−14.25, −0.23)	0.04*	0.78
ALT	14	−12.64 (−29.75, 4.47)	0.13	33	−13.39 (−21.91, −4.88)	0.003*	0.93
ALP	5	11.8 (−9.82, 33.42)	0.20	12	6.42 (−6.35, 19.19)	0.29	0.61
TC	9	−2.89 (−49.40, 43.62)	0.89	17	25 (1.71, 48.28)	0.04*	0.20
HDL	9	8 (0.77, 15.23)	0.03*	17	8.23 (3.95,12.52)	< 0.001*	0.95
TG	9	−58.22 (− 110.6, −5.84)	0.03*	17	−32.65 (− 54.10, −11.19)	0.005*	0.25
LDL	9	−4.89 (−49.80, 40.02)	0.81	16	11.13 (−10.52, 32.77)	0.29	0.43
Vitamin B12	4	206.48 (− 131.01, 543.96)	0.15	12	− 113.33 (−225.72, −0.93)	0.048*	0.01*
Folate	4	0.52 (−5.38, 6.41)	0.80	12	−2.17 (−5.19, 0.86)	0.14	0.33
Ferritin	3	− 177.3 (− 576.75, 222.15)	0.20	11	1.16 (−59.06, 61.39)	0.97	0.02*
Iron	2	19.5 (− 215.56, 254.56)	0.48	5	−26.2 (−65.32, 12.92)	0.14	0.13
TIBC	1	−22	–	4	−95.25 (− 121.79, − 68.71)	0.001*	0.03*
Vitamin D	8	10.74 (−3.13, 24.62)	0.11	15	25.67 (−11.03, 62.37)	0.16	0.54

*Hb* hemoglobin (g/dL), *Plt* platelet (x10^3^g/dL), *WBC* White blood cells (x10^3^g/dL), *FPG* fasting plasma glucose, *HbA1C* hemoglobinA1C, *Cr* creatinine, *AST* Aspartate aminotransferase, *ALT* Alanine aminotransferase, *ALP* alkaline phosphatase, *TC* total cholesterol, *HDL* high-density lipoprotein, *TG* triglyceride, *LDL* low-density lipoprotein, *TIBC* total iron binding capacity

*p < 0.05

When compared between the successful vs. non-successful weight loss groups (between group analysis, Table 3, last column), the successful weight loss group had a significantly larger decreases in vitamin B12 and TIBC (*p* = 0.01 and 0.029, respectively). A significantly greater increase in ferritin was detected in the successful weight loss group (*p* = 0.023).

## Discussions

From this present study, we are able to demonstrate that lower numbers of patients in the successful weight loss group were affected by certain skin signs, namely KP and pebble finger.

Moreover, bariatric surgery provided improvements in metabolic profiles. There were decreases of FPG, HbA1C and TG, and increase in HDL in both groups. Interestingly, only the successful weight loss group exhibited significant decreases of AST and ALT. However, lower level of vitamin B12 and TIBC and an increase in ferritin were detected in the successful group.

Improvements of AN, acrochordons, intertrigo, hidradenitis suppurativa, psoriasis and necrobiosis lipoidica have been reported after bariatric surgery [18]. The result of our study also demonstrates that in patients who achieved successful weight loss, the prevalence of KP and pebble finger is lower. The correlation of AN, KP and pebble fingers with insulin resistance and hyperinsulinemia is well established [3, 5, 7]. Diet control that leads to weight reduction can improve insulin resistance status and lessen AN severity [23]. We speculate that the lower number of KP and pebble fingers in the successful group might result from a greater improvement of insulin resistance after weight reduction in this group.

Alopecia can be associated with nutritional deficiency and an abrupt and massive weight loss [17, 24]. A one-year prospective study showed that 41% of post-bariatric surgery women developed alopecia related to iron and zinc deficiency, and that zinc supplementation could improve the condition [24]. A significantly higher prevalence of alopecia was observed in successful weight loss patients in our study. Though after adjustment with the patients’ factors, it became statistically non-significant, the OR and upper 95%CI were still approximately the same in the adjusted and the unadjusted models, indicating a higher risk of alopecia even after adjustment for patient factors. All patients included in this study were routinely prescribed with nutritional supplements after the surgery (multivitamins1–2 tablets, calcium 1000–2000 mg per day, vitamin D 40,000 units per week and B12 1000 mcg injection every 3 months).

Improvements in laboratory metabolic profiles are well demonstrated in our study. A systematic review and meta-regression demonstrated that a reduction of BMI by 1.6 kg/m^2^ in children can decrease TG by 16 mg/dL and increase HDL by 1.7 mg/dL. On the other hand, other blood profiles, i.e. TC, low-density lipoproteins (LDL), AST, ALT, gamma-glutamyl transferase (GGT), FPG, and HbA1c, are not significantly correlated with degree of weight loss [25–28]. In addition, a long-term study in adult population has shown that weight loss after bariatric surgery can lead to remission and prevention of type 2 DM, dyslipidemia and hypertension [28]. These are consistent with our data. In this present study, TG and HDL-c were shown to improve significantly in post-bariatric surgery patients regardless of their successful weight loss status. Moreover, we were also able to demonstrate significant improvements in AST and ALT profiles only in the individuals achieving successful weight loss. These data suggest that achieving successful weight control might additionally, improve the liver condition.

Nutritional deficiency is a well-established complication of bariatric surgery [17, 20] with vitamin B12 deficiency being the most common [20]. Clinical skin signs associated with vitamin B12 deficiency are cheilitis, glossitis, hair depigmentation, and diffuse or symmetric hyperpigmentation [20]. In this study, we did not find any skin signs of B12 deficiency in our patients. Moreover, none of the patients showed levels below the normal cut-off point (< 148 pmol/l). However, statistically significantly decreased levels of vitamin B12 in the successful weight loss group was observed. This emphasizes that blood profiles might be a better early predictor to evaluate nutritional status, compared to the skin signs or deficiency determined by cut off points alone. Interestingly, an increase in ferritin was detected in the successful group. We speculate that this might be due to a better compliance in taking the supplements of the patients in the group. Further studies are needed to confirm this finding.

The limitations of our study include its cross-sectional nature. The skin examination is conducted only once. Therefore, we cannot evaluate skin improvements from the baseline, only the prevalence at the point of the examination can be obtained. Certain skin signs have been reported to get better after weight reduction. From our study, we were able to identify that there were less patients with KP and pebble finger in the successful group. These results were in keeping with the fact that weight reduction may improve the adverse skin changes. However, long-term prospective studies are needed to demonstrate changes through the course and at later times after the surgery.

## Conclusions

Weight loss after bariatric surgery provides an improvement in metabolic profiles. Furthermore, less patients with successful weight loss had certain skin findings. Importantly, nutritional supplement is necessary for post bariatric surgery individuals, since some skin signs might be late presentations indicating a greater degree of severity.

## Data Availability

The data that support the findings of this study are available from the corresponding author upon reasonable request.

## Abbreviations

BaS: Bariatric surgery
EBWL: Excessive body weight loss
KP: Keratosis pilaris
AST: Aspartate aminotransferase
ALT: Alanine aminotransferase
BMI: Body mass index
AN: Acanthosis nigricans
DM: Diabetes mellitus
FPG: Fasting plasma
HbA1C: Hemoglobin A1C
TG: Triglyceride
HDL: High-density lipoproteins
TIBC: Total iron binding capacity
LDL: Low-density lipoproteins
GGT: Gamma-glutamyl transferase
